# Supplementary material for: Factors Associated with COVID-19 Vaccination Hesitancy and Most Frequently Vaccinated Status in a Japanese Population-Based Sample
Source: Vaccines (Basel). 2024 May 7;12(5):501. doi: 10.3390/vaccines12050501 (PMC11125889; doi:10.3390/vaccines12050501)
Supplement: Supplementary file 1 [file vaccines-12-00501-s001.zip › vaccines-2956054-supplementary.pdf]

		Options												
	Sex	1=Male	2=Female											
	Age													
	Age ID (age group)	1=under 12	2=12-19	3=20-24	4=25-29	5=30-34	6=35-39	7=40-44	8=45-49	9=50-54	10=55-59	11=over 60		
	Prefecture	1=北海道	2=青森県	3=岩手県	4=宮城県	5=秋田県	6=山形県	7=福島県	8=茨城県	9=栃木県	10=群馬県	11=埼玉県	12=千葉県	13=東京都
		14=神奈川県	15=新潟県	16=富山県	17=石川県	18=福井県	19=山梨県	20=長野県	21=岐阜県	22=静岡県	23=愛知県	24=三重県	25=滋賀県	26=京都府
		27=大阪府	28=兵庫県	29=奈良県	30=和歌山県	31=鳥取県	32=島根県	33=岡山県	34=広島県	35=山口県	36=徳島県	37=香川県	38=愛媛県	39=高知県
		40=福岡県	41=佐賀県	42=長崎県	43=熊本県	44=大分県	45=宮崎県	46=鹿児島県	47=沖縄県					
	Area	1=Hokkaido	2=Tohoku	3=Kanto	4=Tyubu	5=Kinki	6=Tyuugoku	7=Shikoku	8=Kyusyu			2=Hokkaido	3=Tohoku	4=Kanto
	Married (marital status) *Q2 is also about the marital status	1=Not married (including divorced/widowed cases)	2= Married											
	HIncome (household's income)	1=Under 2 million	2=2-4 million	3=4-6 million	4=6-8 million	5=8-10 million	6=10-12 million	7=12-15 million	8=15-20 million	9=over 20 million	10=Don't know	11=No answer		
	PIncome(Personal income)	1=Under 2 million	2=2-4 million	3=4-6 million	4=6-8 million	5=8-10 million	6=10-12 million	7=12-15 million	8=15-20 million	9=over 20 million	10=Don't know	11=No answer		
	Job	1=Public survant	2=Buisiness person/board member	3=Employee (clerical work)	4=Employee (technical)	5=Employee (others)	6=Self-employed buisiness	7=Freelance	8=Housewife (husband)	9=Part-time job	10=Student	11=others	12=Disemployment	
Q1	Including you, how many people constantly live in your household (including children and adults)?	1_N number	2=Refusal											
Q2	Marital status	1=Have never been married	2 = Married/co-habiting	3=Divorced	4=Widowed									
Q3	What is the last school you graduated from (or is currently attending)?	1=Juniour high school	2=High school	3=Specialized vacational high school	4=Two-year colledge	5=University	6=Graduate school							
Q4	How has your household's economic situation changed during the past year?	1=Improved a lot	2=Improved a little	3=Stayed about the same	4=Worsened a little	5=Worsened a lot	6=Don't know							
Q5	The following questions ask you about your experiences during the coronavirus pandemic	1=Yes	2 =No											
	1. Do you worry about catching coronavirus?													
	2. Do you take precautions to avoid catching coronavirus such as wearing a mask?													
	3. Have you been vaccinated against coronavirus?													
	4. Have you had coronavirus?													
Q6	The following questions ask you about your experiences during the coronavirus pandemic													
	1. How many times have you been vaccinated against coronavirus?	N time(s)												
	2. How many times have you had coronavirus?	N time(s)												
Q7	In general, would you say your health is....	1=Very good	2=Good	3=Fair	4=Poor	5=Very poor	6=Don't know							
Q8	Is there anyone …													
	1. who you can really count on to listen to you when you need to talk?	1=Yes	2=No	3=Don't know										
	2. who you can really count on to help you out in a crisis/in your most difficult moments?													
	3. who you can totally be yourself with?													
	4. who you feel appreciates you as a person?													
	5. who can comfort you when you are very upset?													
Q9	Loneliness (Igarashi et al., 2019)													
	1. How often do you feel that you lack companionship?													
	2. How often do you feel left out?	1=Hardly ever	2=Some of the time	3=Often										
	3. How often do you feel isolated from others?													
Q10	How much stress do you feel in your daily life?	1=A little	2=A moderate amount	3=A high amount	4=A severe amount	5=Don't feel stress								
Q11	Tobacco Use – Do you smoke tobacco or electronic cigarettes?	1. Yes, ____ cigarettes a day (please write the number of cigarettes)	2. Yes, but not every day	3. No, I stopped smoking	4. No, I never smoked									
	If Q11=3, Q12 was shown													
Q12	Please state the year_____ and month_____ you stopped smoking	N year(s)	N month(s)											
Q13	The following questions refer to drinking alcohol. Please respond to each question.	1=Yes	2=No											
	1. Have you ever felt you should cut down on your drinking?													
	2. Have people annoyed you by criticizing your drinking?													
	3. Have you ever felt bad or guilty about your drinking?													
	4. Have you ever had a drink first thing in the morning to steady your nerves or to get rid of a hangover (eye opener)?													
Q14	Overall in the last 30 days, how much of a problem did you have with sleeping, such as falling asleep, waking up frequently during the night, or waking up too early in the morning?	1=None	2=Middle problems	3=Moderate problems	4=Severe problems	5=Extreme problems								
Q15	Have you ever thought of taking your life, even if you would not really do it?	1=Yes	2=No	3=Do not understand/does not apply										
Q16	Was this	1=Before the coronavirus	2=During the coronavirus pandemic	3=Both before the pandemic began and during the pandemic										
Q17	Have you ever had any of the following health conditions that were diagnosed by a doctor or other health professional? (multiple chlice allowed)													
	1. High blood pressure													
	2. Stroke(e.g. brain hemorrhage, cerebral infarction)													
	3. Heart disease													
	4. Diabetes													
	5. Respiratory disease (e.g. pneumonia, bronchitis)													
	6. Gastrointestinal, liver, or gallbladder disease													
	7. Kidney or prostate gland disease													
	8. Cancer (malignant tumor)													
	9. Other – please state_____													
	10. I haven't received any of them													

Q18	Gambling behaviour (Tanaka, 2010, Ministry of health labor and welfare grants-in-aid for scientific research report (in Japanese))	1=Yes	2=No					
	1. Have you ever gambled before?							
	2. Have you ever returned to gambling on another day to win back the money you lost?							
	3. Have people criticised your gambling?							
	4. Have you ever argued with people you live with over how you handle money for gambling?							
	5. Have you ever borrowed from someone and not paid them back as a result of your gambling?							
Q19	Have you ever borrowed money to gamble or pay gambling debts from any of the following items?	1=Yes	2=No					
	1. Household money							
	2. Loan sharks							
	3. Banks or loan companies							
Q20	Have you ever thought about what age you will live to?	1=Yes	2=No					
Q21	What do you think, up to what age will you live?	N year(s)						
Q22	How often in the past week have you eaten…	1=Daily/almost daily	2=several times per week	3=Once a week	4=Less than onece a week	5=Don't know		
	1. Fresh vegetables (not including potatoes)							
	2. Fresh fruit							
Q23	BMI							
	1. How tall are you without shoes?	N cm						
	2. What is your weight (without clothes and shoes)?	N kg						
Q24	Do you have a sense of purpose in life ("ikigai")?	1=Yes	2=No	3=Don't know				
Q25	Please indicate if you own any pets (multiple choice allowed)							
	Dog							
	Cat							
	Other pets							
	I do not have a pet							
Q26	PHQ9							
	Over the last 2 weeks, how often have you been bothered by any of the following problems?	1=Not at all	2=Several days	3=More than half the days	4=Nearly everyday			
	1. Little interest or pleasure in doing things							
	2. Felling down, depressed, or hopeless							
	3. Trouble falling/staying asleep, sleeping too much							
	4. Feeling tired or having little energy							
	5. Poor appetite or overeating							
	6. Feeling bad about yourself or that you are a failure or have let yourself or your family down							
	7. Trouble concentrating on things, such as reading the newspaper or watching television							
	8. Moving or speaking so slowly that other people could have noticed. Or the opposite; being so fidgety or restless that you have been moving around a lot more than usual.							
	9. Thoughts that you would be better off dead or of hurting yourself in some way.							
Q27	If you checked off any problem on this questionnaire so far, how difficult have these problems made it for you to do your work, take care of things at home, or get along with other people?	1=Not difficult at al	2=Somewhat difficult	3=Very difficult	4=Extremely difficult			
Q28	GAD7							
	Over the last 2 weeks, how often have you been bothered by any of the following problems?							
	1. Feeling nervous, anxious, or on edge	1=Not at all sure	2=Several days	3=Over half the days	4=Nearly everyday			
	2. Not being able to stop or control worrying							
	3. Worrying too much about different things							
	4. Trouble relaxing							
	5. Being so restless that it's hard to sit still							
	6. Becoming easily annoyed or irritable							
	7. Feeling afraid as if something awful might happen							
Q29	If you checked off any problems, how difficult have these made it for you to do your work, take care of things at home, or get along with other people?	1=Not difficult at al	2=Somewhat difficult	3=Very difficult	4=Extremely difficult			
Q30	ASRS	1=Never	2=Rarely	3=Sometimes	4=Often	5=Very often		
	1. How often do you have trouble wrapping up the final details of a project, once the challenging parts have been done?							
	2. How often do you have difficulty getting things in order when you have to do a task that requires organization?							
	3. How often do you have problems remembering appointments or abligations?							
	4. When you have a task that requires a lot of thought, how often do you avoid or delay getting started?							
	5. How often do you fidget or squirm with your hands or feet when you have to sit down for a long time?							
	6. How often do you feel overly active and compelled to do things, like you were driven by a motor?							

Q31	BAP (Sakai et al., 2014)	1=Very rarely	2=Rarely	3=Occasionally	4=Somewhat often	5=Often	6=Very often	
	1. I like being around other people							
	2. I find it hard to get my words out smoothly							
	3. I am comfortable with unexpected changes in plans							
	4. It's hard for me to avoid getting sidetracked in conversation							
	5. I would rather talk to people to get information than to socialize							
	6. People have to talk me into trying something new							
	7. I am "in-tune" with the other person during conversation							
	8. I have to warm myself up to the idea of visiting an unfamiliar place							
	9. I enjoy being in social situations							
	10. My voice has a flat or monotone sound to it							
	11. I feel disconnected or "out of sync" in conversations with others							
	12. People find it easy to approach me							
	13. I feel a strong need for sameness from day to day							
	14. People ask me to repeat things I've said because they don't understand							
	15. I am flexible about how things should be done							
	16. I look forward to situations where I can meet new people							
	17. I have been told that I talk too much about certain topics							
	18. When I make conversation it is just to be polite							
	19. I look forward to trying new things							
	20. I speak too loudly or softly							
	21. I can tell when someone is not interested in what I am saying							
	22. I have a hard time dealing with changes in my routine							
	23. I am good at making small talk							
	24. I act very set in my ways							
	25. I feel like I am really connecting with other people							
	26. People get frustrated by my unwillingness to bend							
	27. Conversation bores me							
	28. I am warm and friendly in my interactions with others							
	29. I leave long pauses in conversation							
	30. I alter my daily routine by trying something different							
	31. I prefer to be alone rather than with others							
	32. I lose track of my original point when talking to people							
	33. I like to closely follow a routine while working							
	34. I can tell when it is time to change topics in conversation							
	35. I keep doing things the way I know, even if another way might be better							
	36. I enjoy chatting with people							
Q32	PRIME Screen-Reivesed (Kobayashi et al., 2008) The original PRIME-screen has 12 items. But Kobayashi et al., (2009) excluded the last item from the rating because only this item did not refer to attenuated positive symptoms. So, the PS-R has 11 items.							
	1. I think that I have felt that there are odd or unusual things going on that I can't explain.	1=Definitely disagree	2=Somewhat disagree	3=Slightly disagree	4=not sure	5=Slightly agree	6=Somewhat agree	7=Definitely agree
	2. I think that I might be able to predict the future.							
	3. I may have felt that there could possibly be something interrupting or controllingmy thoughts, feelings, or actions.							
	4. I have had the experience of doing something differently because of my superstitions.							
	5. I think that Imay get confused at timeswhether something I experience or perceive may be real or may be just part of my imagination or dreams.							
	6. I have thought that it might be possible that other people can read my mind, or that I can read other's minds							
	7. I wonder if people may be planning to hurt me or even may be about to hurt me.							
	8. I believe that I have special natural or supernatural gifts beyond my talents and natural strengths.							
	9. I think I might feel like my mind is "playing tricks" on me.							
	10. I have had the experience of hearing faint or clear sounds of people or a person mumbling or talking when there is no one near me.							
	11. I think that Imay hearmy ownthoughts being said out loud.							
Q33	PRIME Screen-Reivesed (Kobayashi et al., 2008)	less than 1 month	between 1 month and 1 year	more than 1 year				
Q34	Have you experienced any of the following events over the past year? (multiple choice allowed)							
	1. I started a new job							
	2. I quit my job or retired							
	3. I got married							
	4. I got divorced							
	5. I started living alone							
	6. I became better off financially							
	7. I became worse off financially							
	8. A new son/daughter/grandchild/great-grandchild was born							
	9. I lost my spouse							
	10. A family member or close friend or relative passed away							
	11. I suffered a serious illness							
	12. I started caring for sick family members							
	13. Other ( )							
	14. No major changes							
Q35	How often do you laugh out loud?	1=Almost every day	2=1-5 days a week	3=1-3 times a month	4=Never/almost never			
Q36	Financial debts	1=Yes	2=No					
	1. Do you have financial debts?							
	2. Are you worried about your financial debts?							
	If Q36=1 Q37 was shown							
Q37	How long do you think it will take you to pay off your financial debts?	1=Less than 1 year	2=1-2 years	3=3-5 years	4=6-10 years	5=More than 10 years		
Q38	Do you have a disability?	1=Yes	2=No					
